# SARS-CoV-2 Variants, RBD Mutations, Binding Affinity, and Antibody Escape

**DOI:** 10.3390/ijms222212114

**Published:** 2021-11-09

**Authors:** Lin Yang, Jiacheng Li, Shuai Guo, Chengyu Hou, Chenchen Liao, Liping Shi, Xiaoliang Ma, Shenda Jiang, Bing Zheng, Yi Fang, Lin Ye, Xiaodong He

**Affiliations:** 1 National Key Laboratory of Science and Technology on Advanced Composites in Special Environments, Center for Composite Materials and Structures, Harbin Institute of Technology, Harbin 150080, China; 19B918053@stu.hit.edu.cn (J.L.); 20B918032@stu.hit.edu.cn (S.G.); shiliping@hit.edu.cn (L.S.); maxiaoliang@hit.edu.cn (X.M.); 20S118122@stu.hit.edu.cn (S.J.); 2 School of Aerospace, Mechanical and Mechatronic Engineering, The University of Sydney, Sydney, NSW 2006, Australia; lin.ye@sydney.edu.au; 3 School of Electronics and Information Engineering, Harbin Institute of Technology, Harbin 150080, China; houcy@hit.edu.cn (C.H.); 1170500818@stu.hit.edu.cn (C.L.); 4 Key Laboratory of Functional Inorganic Material Chemistry (Ministry of Education) and School of Chemistry and Materials Science, Heilongjiang University, Harbin 150001, China; zhengbing@hlju.edu.cn; 5 Mathematical Science Institute, The Australian National University, Canberra, ACT 0200, Australia; yi.fang3@gmail.com; 6 Shenzhen STRONG Advanced Materials Research Institute Co., Ltd., Shenzhen 518035, China

**Keywords:** SARS-CoV-2, variants, RBD, mutations, antibody

## Abstract

Since 2020, the receptor-binding domain (RBD) of the spike protein of the novel severe acute respiratory syndrome coronavirus 2 (SARS-CoV-2) has been constantly mutating, producing most of the notable missense mutations in the context of “variants of concern”, probably in response to the vaccine-driven alteration of immune profiles of the human population. The Delta variant, in particular, has become the most prevalent variant of the epidemic, and it is spreading in countries with the highest vaccination rates, causing the world to face the risk of a new wave of the contagion. Understanding the physical mechanism responsible for the mutation-induced changes in the RBD’s binding affinity, its transmissibility, and its capacity to escape vaccine-induced immunity is the “urgent challenge” in the development of preventive measures, vaccines, and therapeutic antibodies against the coronavirus disease 2019 (COVID-19) pandemic. In this study, entropy–enthalpy compensation and the Gibbs free energy change were used to analyze the impact of the RBD mutations on the binding affinity of SARS-CoV-2 variants with the receptor angiotensin converting enzyme 2 (ACE2) and existing antibodies. Through the analysis, we found that the existing mutations have already covered almost all possible detrimental mutations that could result in an increase of transmissibility, and that a possible mutation in amino-acid position 498 of the RBD can potentially enhance its binding affinity. A new calculation method for the binding energies of protein–protein complexes is proposed based on the entropy–enthalpy compensation rule. All known structures of RBD–antibody complexes and the RBD–ACE2 complex comply with the entropy–enthalpy compensation rule in providing the driving force behind the spontaneous protein–protein docking. The variant-induced risk of breakthrough infections in vaccinated people is attributed to the L452R mutation’s reduction of the binding affinity of many antibodies. Mutations reversing the hydrophobic or hydrophilic performance of residues in the spike RBD potentially cause breakthrough infections of coronaviruses due to the changes in geometric complementarity in the entropy–enthalpy compensations between antibodies and the virus at the binding sites.

## 1. Introduction

The coronavirus disease 2019 (COVID-19) has spread worldwide, with more than 230 million confirmed cases, and has led to an ongoing pandemic. In response to this once-in-a-century, sustained, worldwide pandemic, unprecedented amounts of funds and manpower have been invested to develop vaccines against the disease by governments, corporations, university research groups, and international health organizations [1]. Thanks to the rapid development of vaccine technology in recent years [1], six vaccines approved by the World Health Organization (WHO) for emergency use have been massively employed in global vaccination programs to immunize the population against COVID-19 infection, while dozens of other vaccine candidates are being prepared for Phase III trials. By September 2020, about 20 countries had vaccinated over 70% of their populations, and the best vaccination rates in the world are not only dominated by small countries. Almost all existing vaccines have been developed based on the initial SARS-CoV-2 strain that was originally identified in China, or on the genetic sequence data that enable vaccine-induced antibodies to stop the original SARS-CoV-2 strain from spreading in a fully vaccinated population. Indeed, the original SARS-CoV-2 strain is disappearing in countries with the best vaccination rates. However, the SARS-CoV-2 variants have almost replaced the initial SARS-CoV-2 strain and are spreading in these countries, according to GISAID data [2,3,4]. At present, in Japan, the proportion of cases infected with the Delta variant is close to 65%, and it is constantly increasing; in Singapore, infection by the Delta strain has accounted for about 95% of all infections in the region; in Israel, about 90% of new infections are likely caused by the Delta variant; in the United States, the Delta virus infection rate has reached 83% among the newly infected people tested; in the United Kingdom, the proportion of Delta infection samples in the last four weeks before the writing of this paper was as high as 99%.

Four Variants of Concern (VOCs) and five Variants of Interest (VOIs) are recognized by the WHO. The increased transmissibility and breakthrough infections caused by the VOCs are believed to be due to mutations in the structure of the spike (S) proteins [5]. There have been a number of missense mutations observed in the receptor-binding domain (RBD) of the SARS-CoV-2 S protein, which have presented in one or more of the VOCs, including the N440K, G446V, L452R, Y453F, E484Q, F490S, N501Y, N501S, E484K, and K417N [5,6,7,8], most of which are located at the RBD–ACE2 interface. Understanding the physical mechanism responsible for the mutation-induced changes in the RBD’s binding affinity with the receptor angiotensin converting enzyme 2 (ACE2) and antibodies is the “urgent challenge” in the development of preventive measures, vaccines, and therapeutic antibodies against the COVID-19 pandemic [8,9,10,11,12,13,14,15]. A key question is whether existing COVID-19 vaccine-induced antibodies can protect against the infection or diseases from these SARS-CoV-2 variants. Most vaccine-induced antibodies neutralize the SARS-CoV-2 variants via the protein–protein docking between the CoV S RBD and the antibodies [1]. The key to vaccine-induced immunity is the ability of the induced antibodies to specifically bind to the SARS-CoV-2 with a strong binding affinity. The complex structures of the antibodies bound to the SARS-CoV-2 S RBD have been experimentally determined by using cryo-electron microscopy (cryo-EM) and X-ray diffraction [12,16,17,18,19,20,21,22,23,24,25,26,27,28]. In the intracellular environment and in an extracellular medium, protein–protein docking is usually a spontaneous contact of high specificity established between two or more specific protein molecules; erroneous protein–protein docking rarely occurs [29]. The specific binding affinity of protein–protein docking is therefore considered one of the miracles of nature [30,31].

As a typical spontaneous reaction, protein–protein docking (i.e., protein–ligand binding) must release Gibbs free energy as it proceeds, namely, the change of Gibbs free energy $\Delta G_{bin}$ during the binding process must be negative. The Gibbs free energy is defined as follows:

G=H−TS

where T is the temperature, S is the entropy, and H is the enthalpy. The Gibbs free energy equation can describe the spontaneity of a reaction, and changing the values of enthalpy and entropy affect the spontaneity [32]. When using the change of Gibbs free energy to assess the spontaneity of protein–protein docking, it is important to emphasize that the definition of enthalpy and entropy apply to the whole system of proteins and the surrounding water molecules [32]. Water molecules (i.e., hydration shell) are able to saturate the hydrogen (H)-bond formations of the hydrophilic groups on the protein surface before protein–protein docking [33,34,35] because water molecules have strong hydrogen bonding interactions [35,36,37,38,39,40,41,42,43]. The hydration shell (i.e., hydration layer) around a protein has been experimentally found to possess dynamics that is distinct from the bulk water at a distance of 1 nm, and water molecules slow down greatly when they enter the hydration shell of a protein [33,44]. The hydrophilic groups of the protein surface are normally hydrogen bonded with the surrounding water molecules, thereby preventing the surface H-bond donors of a protein from randomly hydrogen-bonding with the H-bond acceptors of another protein; namely, the erroneous protein–protein docking in unsaturated aqueous solutions is prevented [33,34,35,45]. Thus, protein–protein docking starts from the long-range attraction between the hydration shells of proteins.

**Figure 1:**
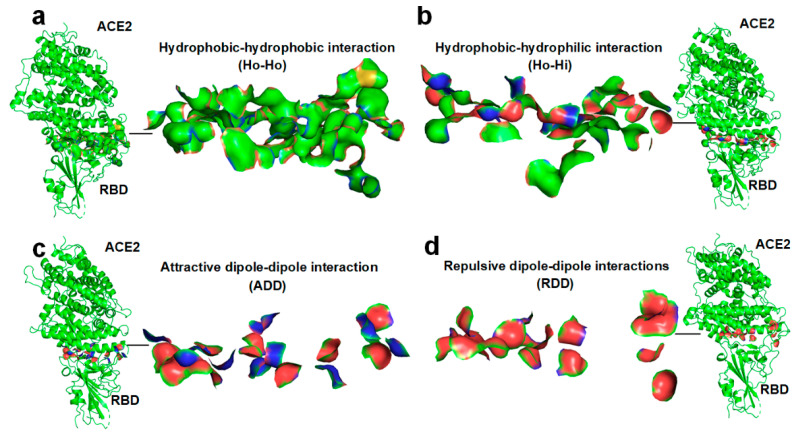
Four types of surface interactions between SARS-CoV-2 S RBD and ACE2 at the interface of the complex. (**a**) The hydrophobic–hydrophobic (Ho-Ho) interactions at the RBD–ACE2 complex interface. (**b**) The hydrophobic–hydrophilic (Ho-Hi) interactions at the RBD–ACE2 complex interface. (**c**) The attractive dipole–dipole (ADD) interactions at the interface of the RBD–ACE2 complex. (**d**) The repulsive dipole–dipole (RDD) interactions at the interface of the RBD–ACE2 complex. The hydrophobic surface areas are highlighted in green and yellow, the hydrophilic surface areas are highlighted in red and blue.

Using biophysical assays, Wrapp et al. found that the binding affinity of SARS-CoV-2 S RBD to ACE2 is more than 10-fold higher than that of the corresponding spike protein of SARS-CoV to the host cell receptor [11]. The hydrophobic interaction between SARS-CoV-2 S and the ACE2 protein is found to be significantly greater than that between SARS-CoV S and ACE2 [46], revealing the resource of the outstanding SARS-CoV-2-S-ACE2 binding affinity to some extent [11]. The binding affinity between two proteins can be attributed to the long-range hydrophobic effect among the hydrophobic surface areas of the two proteins at the binding sites, which enable the hydrophobic surface areas to fully collapse together in between the two proteins of the complex (see Figure 1a) [46,47,48,49]. Moreover, the assembling process of tertiary structures into a quaternary structure is essentially the same as that of protein–protein docking. The folding of protein quaternary structures is found to be guided by the entropy–enthalpy compensations between the binding sites of protein subunits, according to the Gibbs free energy equation that is verified by the bioinformatic analyses of a dozen structures of dimers [50]. More specifically, entropy increments caused by hydrophobic surface areas collapse in between protein subunits, thus compensating for the increment of enthalpy caused by H-bond formation between the protein subunits [50].

At the interface of a protein–protein complex, interactions among the surface areas of proteins can be classified into four types: hydrophobic–hydrophobic (Ho-Ho) interaction, hydrophobic–hydrophilic (Ho-Hi) interaction, attractive dipole–dipole (ADD) interaction, and repulsive dipole–dipole (RDD) interaction. The four types of surface interactions between the SARS-CoV-2 S RBD and the ACE2 are illustrated in Figure 1 and in the Supplementary Figure S1. As a direct result of a protein–protein docking, the networks of ordered water molecules (i.e., hydration shell) that once covered the protein’s binding site are removed from each protein by the docking action, which changes the entropy. Moreover, at the binding sites, the H-bonds that once connected the water molecules and hydrophilic groups of protein are broken due to the docking. Thereby, all the four types of surface interactions between proteins change the enthalpy and entropy values of the system. Entropy–enthalpy compensation should thus be considered a key mechanism that governs protein–protein docking processes. Note that the hydrophilicity of proteins at the binding site is normally expressed by CO or NH groups at the ends of hydrophilic side chains or the main chain [50]. Therefore, the attractive dipole–dipole interaction can be considered the hydrogen bonding between the CO groups and NH groups of the protein chains (see Figure 1c). The repulsive dipole–dipole interaction can be considered the electrostatic repulsion between negative carbonyl oxygen atoms (see Figure 1d) or between positive amide hydrogen atoms.

**Figure 2:**
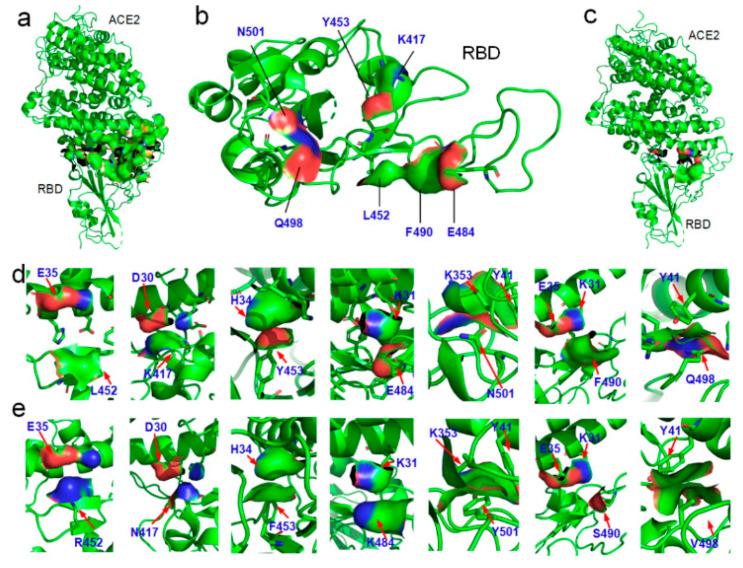
Binding surfaces of the RBD and the ACE2. (**a**) The RBD–ACE2 complex (PDBID: 6LZG). (**b**) The distribution of hydrophobic (green and yellow areas) and hydrophilic (red and blue areas) surface areas at the amino-acid positions L452, K417, Y453, E484, N501, F490, and Q498 of the RBD. (**c**) Ho-Hi surface area interactions in the complex. (**d**) Seven cases of Ho-Hi surface interactions identified at amino-acid positions L452, K417, Y453, E484, N501, F490, and Q498 of the RBD in the RBD–ACE2 complex. (**e**) Transformation from the Ho-Hi surface interactions into Ho-Ho or ADD interactions due to the mutations L452R, K417N, Y453F, E484K N501Y, and F490S.

At the interface of the RBD–ACE2 complex, because of the repulsion between the hydrophobic groups and the hydrophilic groups in water, when a local hydrophobic surface area of the RBD attaches to another hydrophilic surface area of the ACE2, the interactions play a role in decreasing the binding affinity between the two proteins. At the interface, seven cases of the Ho-Hi surface interactions are identified at the amino-acid positions L452, K417, Y453, E484, N501, F490, and Q498 of the RBD, as illustrated in Figure 2. Surprisingly, the mutations of L452R, K417N, Y453F, E484K N501Y, and F490S transform the Ho-Hi interactions into Ho-Ho or ADD interactions at these positions, thus increasing the binding affinity with ACE2. This would lead the RBD–ACE2 binding affinity of the Alpha variant and the Delta variant with ACE2 to be obviously bigger than that of the original SARS-CoV-2. Although the RBD mutation at amino-acid position 498 is not a missense mutation that presented in one or more of the VOCs, the RBD mutation such as Q498A has also been reported [51,52,53].

## 2. Results

### 2.1. Theory of the Spontaneous Nature of Protein–Protein Docking

Entropy–enthalpy compensation and the Gibbs free energy change can be used to analyze the spontaneous nature of protein–protein docking. The change of Gibbs free energy $\Delta G_{bin}$ must be negative for a spontaneous protein–protein docking process in aqueous solutions. If the binding energies of the Ho-Ho, Ho-Hi, ADD, and RDD interactions among surface areas can be separately calculated, the binging energies of protein–protein complexes can be easily calculated based on the entropy–enthalpy compensation rule.

### 2.2. Docking between Hydrophobic Surface Areas

A characteristic of hydration shell water molecules is that their hydrogen bonding network is much more ordered than that of free liquid water molecules; that is, their entropy is lower (less entropy in the system) [54,55,56,57]. The ordered water molecules are fixed in these water cages surrounding the hydrophobic areas that drive the hydrophobic collapse of the hydrophobic surface areas between proteins, thereby rearranging the ordered water molecules into free liquid water molecules. Experimental results show that water molecules slow down greatly when they encounter the hydrophobic areas of a protein, and the speed is reduced by 99% [33]. Thus, the standard molar entropy of water within the ordered cages around the nonpolar surface (i.e., hydration shells) is approximately equal to the standard molar entropy of solid water, and that is about 41 J/mol/K. The standard molar entropy of liquid water is about 70 J/mol/K [58,59]. Therefore, moving an ordered water molecule to free liquid results in an entropy difference ∆S of about 29 J/mol/K. At the human body temperature of T = 309K, increment entropy is T∆S = 8961 J/mol for one removed hydration shell water molecule. The surface density of ordered water molecules surrounding the hydrophobic surfaces areas is about 2 water molecules per 10 Å^2^ [33]. At the human body temperature, the docking-induced hydrophobic interaction between two 10 Å^2^ hydrophobic surfaces areas can expel approximately 4 ordered water molecules from the hydration shell of the hydrophobic regions into a liquid water solvent; the entropy increment for the Ho-Ho binding is about 35,800 J/mol, that is, the $\Delta G_{bin}^{Ho\text{-}Ho}$ is about −35,800 J/mol.

### 2.3. Docking between the Hydrophobic Surface Area and the Hydrophilic Surface Area

In the case of a hydrophobic surface area attaching to a hydrophilic surface area at the interface of a protein–protein complex, the hydrophobic surface area loses its hydration shell (i.e., ordered water molecules) due to the docking, whereas the hydrophilic surface area loses its hydrogen-bonded water molecules due to the docking. The hydrophilicity of hydrophilic side chains or main chain is normally expressed by CO or NH groups at their ends. These hydrophilic CO and NH groups are hydrogen-bonded with the surrounding water molecules in a hydration shell before the docking. The binding energy of the H-bond between the NH group and a water molecule is about −7.65 kcal/mol (32,000 J/mol), and the binding bond energy of the H-bond between the CO group and a water molecule is about −4.66 kcal/mol (19,479 J/mol) [38,39,40,41,42]. Note that after a water molecule loses its hydrogen bonding with the hydrophilic group of a protein, other water molecules are able to saturate the H-bond formations of the water molecule in an aqueous solution as part of the compensation for the enthalpy change, taking the average as about ΔH = 12,000 J/mol per removed hydrogen-bonded water molecule (i.e., an H-bond) from a hydrophilic surface area. The surface density of hydrogen bonds between water molecules and the hydrophilic groups is about 2 hydrogen bonds per 10 Å^2^. Thus, the docking-induced attachment between a 10 Å^2^ hydrophobic surface area and a 10 Å^2^ hydrophilic surface area can expel approximately 2 ordered water molecules from the hydration shell, and break 2 H-bonds between the protein surface hydrophilic groups and the water molecules. The entropy increment is about 17,900 J/mol and the enthalpy increment is about 24,000 J/mol. As a result, the $\Delta G_{bin}^{Ho\text{-}Hi}$ is about 6100 J/mol.

### 2.4. Docking-Induced Attractive Dipole–Dipole Interaction

Intermolecular H-bonds in protein–protein complexes are normally the dipole–dipole attractions between the CO groups and NH groups of the protein chains, acting as the pairing of the H-bond donors and acceptors [60]. The binding energy (H-bond energy) of an intermolecular H-bond between the NH group and the CO group is about −3.47 kcal/mol = 14,518 J/mol [38,61]. A protein’s hydrophilic CO or NH groups are normally hydrogen-bonded with the surrounding water molecules in a hydration shell. When an H-bonded water molecule is removed from the CO group or the NH group, the H-bond between them is broken. After a water molecule loses the hydrogen bonding with the hydrophilic group of a protein, other water molecules are able to saturate the H-bond formations of the water molecule. The binding energy of the H-bond between the NH group and a water molecule is about −7.65 kcal/mol, and the binding bond energy of the H-bond between the CO group and a water molecule is about −4.66 kcal/mol [38,39,40,41,42]. Similarly, the average of about ΔH = 12,000 J/mol per removed hydrogen-bonded water molecule is taken from a hydrophilic surface area. In order to form an intarmolecular H-bond between an H-bond donor and an acceptor in the protein–protein docking, two H-bonds between water molecules and the protein′s hydrophilic groups need to break first. The surface density of an intramolecular H-bond on a hydrophilic surfaces area is about 2 intramolecular H-bond per 10 Å^2^. Thus, for the docking-induced attractive dipole–dipole interaction between two 10 Å^2^ hydrophilic surface areas, the Gibbs free energy change is $\Delta G_{bin}^{ADD}=2\times \left(2\times 12,000-14,518.5H\right)\;J/\mathrm{mol}=19,000\;J/\mathrm{mol}$.

### 2.5. Docking-Induced Repulsive Dipole–Dipole Interaction

Taking the average as about ΔH = 12,000 J/mol per removed hydrogen-bonded water molecule from a hydrophilic surface area, the surface density of H-bonds linking water molecules and proteins on hydrophilic surface areas is about 2 H-bonds per 10 Å^2^. Thus, in the case of the docking of the repulsive dipole–dipole between two 10 Å^2^ hydrophilic surface areas, the change in Gibbs free energy is $\Delta G_{bin}^{RDD}=4\times 12,000\;J/\mathrm{mol}=48,000\;J/\mathrm{mol}$.

## 3. Discussion

### 3.1. Verification of the Docking Theory

Experimentally determined structures of RBD–antibody complexes and the RBD–ACE2 complex are stored in the protein data bank (PDB) archives. The corresponding total area for the Ho-Ho, Ho-Hi, ADD, and RDD interactions at the interface of these protein–protein complexes can be easily measured by using these PDB files, denoted as S_Ho-Ho_, S_Ho-Hi_, S_ADD,_ and S_RDD_. Among the four types of interactions between surface areas during the protein–protein docking, only the Ho-Ho interaction causes the $\Delta G_{bin}$ to decrease, whereas the Ho-Hi, the ADD, and the RDD interactions cause the $\Delta G_{bin}$ to increase. Thus, the docking-induced Gibbs free energy change can be calculated as follows:

(1) $$\begin{array}{c} \Delta G_{bin}\;=\Delta G_{bin}^{Ho\text{-}Ho}\;\times \;S_{\mathrm{Ho}\text{-}\mathrm{Ho}\;}+\Delta G_{bin}^{Ho\text{-}Hi}\;\times \;S_{\mathrm{Ho}\text{-}\mathrm{Hi}}+\Delta G_{bin}^{ADD}\times S_{\mathrm{ADD}}\;+\Delta G_{bin}^{RDD}\;\times \;S_{\mathrm{RDD}}\\ \;=-\mathrm{35,800}\;\times \;S_{\mathrm{Ho}\text{-}\mathrm{Ho}}+\mathrm{6100}\;\times \;S_{\mathrm{Ho}\text{-}\mathrm{Hi}}+\;\mathrm{19,000}\times S_{\mathrm{ADD}}\;+\;\mathrm{48,000}\times S_{\mathrm{RDD}}\; \end{array}$$

It should be noted that Equation (1) does not possess an ab initio physico-mathematical derivation. Equation (1) is more appropriate for binding affinity estimation when proteins are in close contact with each other in the complex. For the cases where proteins are not in close contact with each other in the complex, the equation simply cannot be used.

**Figure 3:**
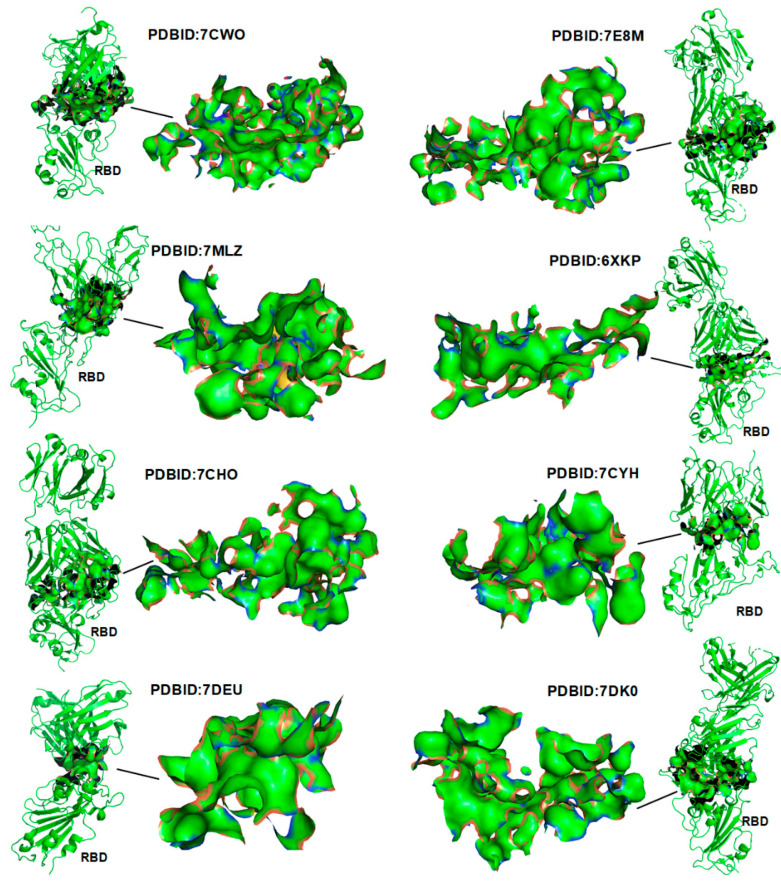
Strong hydrophobic–hydrophobic interactions at the interfaces of 8 RBD–antibody complexes. The distribution of hydrophobic surface areas are highlighted in green and yellow.

**Figure 4:**
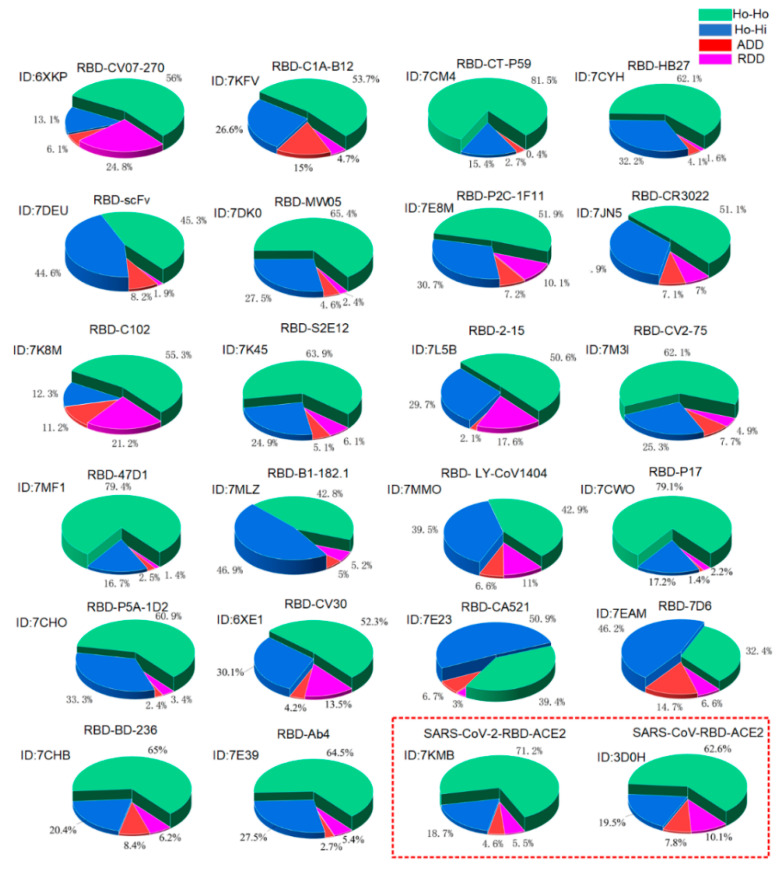
The calculated proportions of SHo-Ho, SHo-Hi, SADD, and SRDD at the interface of RBD–antibody complexes, the RBD–ACE2 complex, and the SARS-CoV-S-RBD–ACE2 complex. The PDBIDs are added in the figure. The results of RBD–ACE2 complex, and the SARS-CoV-S-RBD–ACE2 complex are highlighted with a red dash line.

To realize the spontaneity of protein–protein binding, the total area of the Ho-Ho interaction should normally be larger than that of the three other types of interactions. For example, 22 structures of the RBD–antibody complexes demonstrate the characteristic that the hydrophobic surface areas fully collapsing together in between the proteins of the complexes (see Figure 3 and Figure 4, and the Supplementary Figure S1). Protein–protein docking should be guided by the changing of Gibbs free energy and the entropy–enthalpy compensations at the binding sites according to the Gibbs free energy equation. We calculated the corresponding total S_Ho-Ho_, S_Ho-Hi_, S_ADD,_ and S_RDD_ areas at the interface of the RBD–ACE2 and RBD–antibody protein–protein complexes. The proportions of S_Ho-Ho_, S_Ho-Hi_, S_ADD,_ and S_RDD_ at the interface of the complexes are calculated and illustrated in Figure 4.

The results show that all known structures of the RBD–antibody complexes, the RBD–ACE2 complex (PDBID:7KMB), and the SARS-COV-S-RBD–ACE2 complex (PDBID:3D0H) confirm the entropy–enthalpy compensation rule for providing the driving force behind the spontaneous protein–protein docking, namely, all the calculated values of the $\Delta G_{bin}$ are obviously negative (see Figure 4 and the Supplementary Figure S2). The docking-induced Gibbs free energy change $\Delta G_{bin}$ can be considered the binding affinity. The calculated binding affinity of the SARS-CoV-2-RBD–ACE2 complex is obviously bigger than that of the SARS-CoV-RBD–ACE2 complex (see Figure 4 and the Supplementary Figure S2); it is thus not surprising that SARS-CoV-2 has higher transmissibility than the SARS-CoV [11,46]. In the SARS-CoV-2-RBD–ACE2 complex, it is worth noting the hydrophilic–hydrophobic repulsion between the E35 and L452 located at the edge of the complex interface (see Figure 2d). This means that the distance between the E35 and the L452 is not short enough to destroy the network of ordered water molecules surrounding the L452; that is, the interaction between the E35 and the L452 may not increase entropy, but it does increase enthalpy. However, the change from Leucine (L) to arginine (R) at the amino-acid position 452 due to the mutation L452R introduces two hydrogen bond (i.e., the ADD) interactions between E35 and R452 (see Figure 2d,e). It is worth noting that the arginine (R) residue is the most hydrophilic one among the 20 standard amino acids. The side chain of E is also very hydrophilic, according to the hydrophobicity scales [62]. Both the R and E have strong hydrogen bonding interactions, even in comparison to water molecules, as the charge of their charged side chains is almost equal to that of a water molecule [34,36]. Thereby, the $\Delta G_{bin}$ caused by the R–E ADD interaction may be negligible. The mutation L452R thus increases the binding affinity between the RBD and the ACE2. A recent molecular dynamic study also revealed that hydrophobic interactions are critical to the enhancement of receptor binding and the ability to escape antibody recognition by the RBD of SARS-CoV-2 [60].

### 3.2. Variations of the Binding Affinities of SARS-CoV-2 Due to Mutations

**Figure 5:**
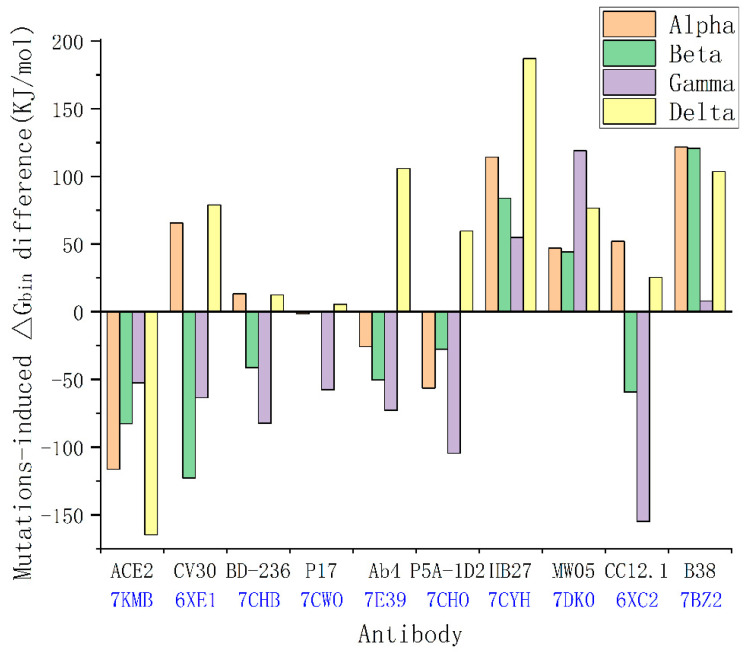
Mutation-induced $\Delta G_{bin}$ difference of the VOCs in the binding with ACE2 as compared to the original SARS-CoV-2, and mutation-induced $\Delta G_{bin}$ difference of the VOCs in the binding with antibodies as compared to the original SARS-COV-2. The corresponding PDB IDs are added in the figure.

When a mutation reverses the hydrophobic or hydrophilic performance of a residue in the RBD, it potentially changes the type of surface interactions in the complexes at this amino acid position. This means that a mutation can change the binding affinity of the RBD with ACE2 or antibodies due to the changes in geometric complementariness for the entropy–enthalpy compensations between proteins. For each variant, the $\Delta G_{bin}$ of the variant′s RBD–ACE2 complex can be calculated by our proposed method (i.e., Equation (1)), which would enable us to compare the binding affinities of the RBD–ACE2 before and after the mutations. The mutation-induced change in the RBD–ACE2 binding affinity can be evaluated from the difference between the calculated $\Delta G_{bin}$ before and after the mutations. We calculated the mutation-induced $\Delta G_{bin}$ difference for all the VOCs in the binding with ACE2 as compared to the original SARS-CoV-2 (see Figure 5). The calculation results show that the Alpha, Beta, Gamma, and Delta variants have increased binding affinity with ACE2 as compared to the original coronavirus.

Using the same method, we evaluated the impact of these mutations on the RBD–antibodies binding affinities. We analyzed the mutation-induced surface hydrophobic and hydrophilic area changes at the binding sites of the complexes of the SARS-CoV-2 variants and 22 existing antibodies [19,62,63,64,65,66], as shown in Figure 5. Surprisingly, the Delta variant can selectively decrease the binding affinity of the antibodies DB-236, CV30, AB4, P17, P5A-1D2, HB27, MW05, CC12.1, and B38, which matches the findings on the Delta variant spreads in the most vaccinated countries (see Figure 5 and Supplementary Figure S2). Through this analysis, we found that the existing mutations have already covered almost all possible detrimental mutations that could result in an increase in transmissibility, and a possible mutation at amino-acid position 498 of the RBD can potentially enhance the binding affinity. In the case of the Alpha variant, the original hydrophilic asparagine (N) at amino-acid position 501 of the RBD was facing a hydrophobic group in the ACE2 at the binding site. The change from asparagine (N) to tyrosine (Y) at amino-acid position 501 increased the hydrophobic attraction and decreased the hydrophilic–hydrophobic repulsion between the two proteins (see Figure 2). Therefore, the change from asparagine (N) to tyrosine (Y) at amino-acid position 501 of the RBD can change the original repulsing relationship between the RBD and ACE2 to an attractive relation between them at amino-acid position 501. Main-chain structures of the SARS-CoV-2 RBD are almost the same as that of the SARS-CoV RBD; this indicates that the spike RBD is of good mechanical stability, promising a stable binding affinity of SARS-CoV-2 S RBD to ACE2 [67,68].

## 4. Materials and Methods

### 4.1. Protein Structures

In this study, many experimentally determined native structures of proteins are used to study the mechanism triggering the docking of SARS-CoV-2 variants to ACE2 and the antibodies. All the three-dimensional (3D) structure data of protein molecules were sourced from the PDB database, including the experimentally determined RBD of SARS-CoV-2 S, ACE2, antibodies, and their complexes. The IDs of these proteins, according to the PDB database, are marked in all the figures. In order to show the distribution of the hydrophobic areas on the surface of the SARS-CoV-2 RBD, ACE2, antibodies, and their complexes at the binding sites in these figures, we used the structural biology visualization software PyMOL to display the protein hydrophobic surface areas. The distribution of the hydrophobic surface areas is highlighted in green (carbon) and yellow (sulfur). The distribution of the hydrophilic surface areas is highlighted in red (oxygen) and blue (nitrogen) since the hydrophilicity of the protein at the binding site is normally expressed by the CO or NH groups at the ends of the hydrophilic side chains or the main chain.

### 4.2. Calculation of Hydrophobic or Hydrophilic Surface Area

According to Equation (1), the binding affinity of RBD and ACE2/antibodies can be characterized by calculating the size of the hydrophobic and hydrophilic contact area in the complex structures. We used the molecular 3D structure display software PyMOL to draw the hydrophobic and hydrophilic surface areas at the binding sites of these complex structures. We calculated the hydrophobic attraction surface areas and the hydrophobic repulsion surface areas involved in the docking among the RBD, ACE2, and antibodies in this study by using integration. With regard to the calculation of S_Ho-Ho_, S_Ho-Hi_, S_ADD,_ and S_RDD_ in the structures of the protein complexes, we sketched the interfaces between the proteins in the complexes as the basis for the calculations of S_Ho-Ho_, S_Ho-Hi_, S_ADD,_ and S_RDD_. 

### 4.3. The Parameters of the Equation (1)

Experimental results show that water molecules slow down greatly when they encounter the hydrophobic areas of a protein, and the speed is reduced by 99% [33]. Therefore, the standard molar entropy of solid water can be used to approximate the standard molar entropy of water within the ordered cages around the protein nonpolar surface (i.e., hydration shells). The hydration shell (i.e., hydration layer) around a protein has been experimentally found to possess dynamics that is distinct from that of bulk water at a distance of 1 nm [33]. Thus, the surface density of ordered water molecules surrounding the hydrophobic surfaces areas (i.e., 2 water molecules per 10 Å^2^) is a conservative estimate. At the human body temperature, the docking-induced hydrophobic interaction between two 10 Å^2^ hydrophobic surface areas may expel approximately more than 4 ordered water molecules from the hydration shell of hydrophobic regions into a liquid water solvent, while the entropy increment for the Ho-Ho binding may be larger than 35,800 J/mol. The surface density of hydrogen bonds between water molecules and the hydrophilic groups (i.e., about 2 hydrogen bonds per 10 Å^2^) was estimated from a survey of the PDB file (PDBID: 6LZG). 

## 5. Conclusions

Based on the entropy–enthalpy compensation rule, a simple measurement calculation of the binding energies of protein–protein complexes was developed. As a typical spontaneous reaction, the RBD–ACE2 docking or the RBD–antibody docking must release Gibbs free energy according the second law of thermodynamics. All the four types of interactions between the surface areas of the docking proteins changed the values of entropy and enthalpy of the system, and affected the spontaneity of protein–protein docking. The entropy–enthalpy compensation involved in the surface area interactions during the docking can be approximately calculated through a survey of these hydrophobic and hydrophilic surface areas, thus enabling the calculation of the binding energy. With these calculations, we showed that all known structures of the RBD–antibody and RBD–ACE2 complexes have strong binding affinities, and enable the hydrophobic surface areas to fully collapse together in between the proteins of the complexes. By means of this analysis, we found that the existing mutations have already covered almost all possible detrimental mutations that could result from the increase in the RBD–ACE2 binding affinity, and a possible mutation at amino-acid position 498 of the RBD can potentially enhance the binding affinity. Variant-induced risk of breakthrough infections among vaccinated people was attributed to the L452R mutations, decreasing the binding affinity of many antibodies. Mutations reversing the hydrophobic or hydrophilic performance of residues in the RBD of coronaviruses potentially cause breakthrough infections due to the changes in geometric complementariness of the entropy–enthalpy compensations between antibodies and the virus at the binding sites. Even though the stronger binding affinity could promote faster entry kinetics for the virus entering the host, this may not equate to the faster spread of the virus in the population; the calculation results match the findings on the Delta variant being more transmissible and spreading more easily in the most vaccinated countries.

## Supplementary Materials

The following are available online at https://www.mdpi.com/article/10.3390/ijms222212114/s1.
