# Supplementary material for: SARS-CoV-2 Variants, RBD Mutations, Binding Affinity, and Antibody Escape
Source: Int J Mol Sci. 2021 Nov 9;22(22):12114. doi: 10.3390/ijms222212114 (PMC8619214; doi:10.3390/ijms222212114)
Supplement: Supplementary file 1 [file ijms-22-12114-s001.zip › ijms-1432036-supplementary.pdf]

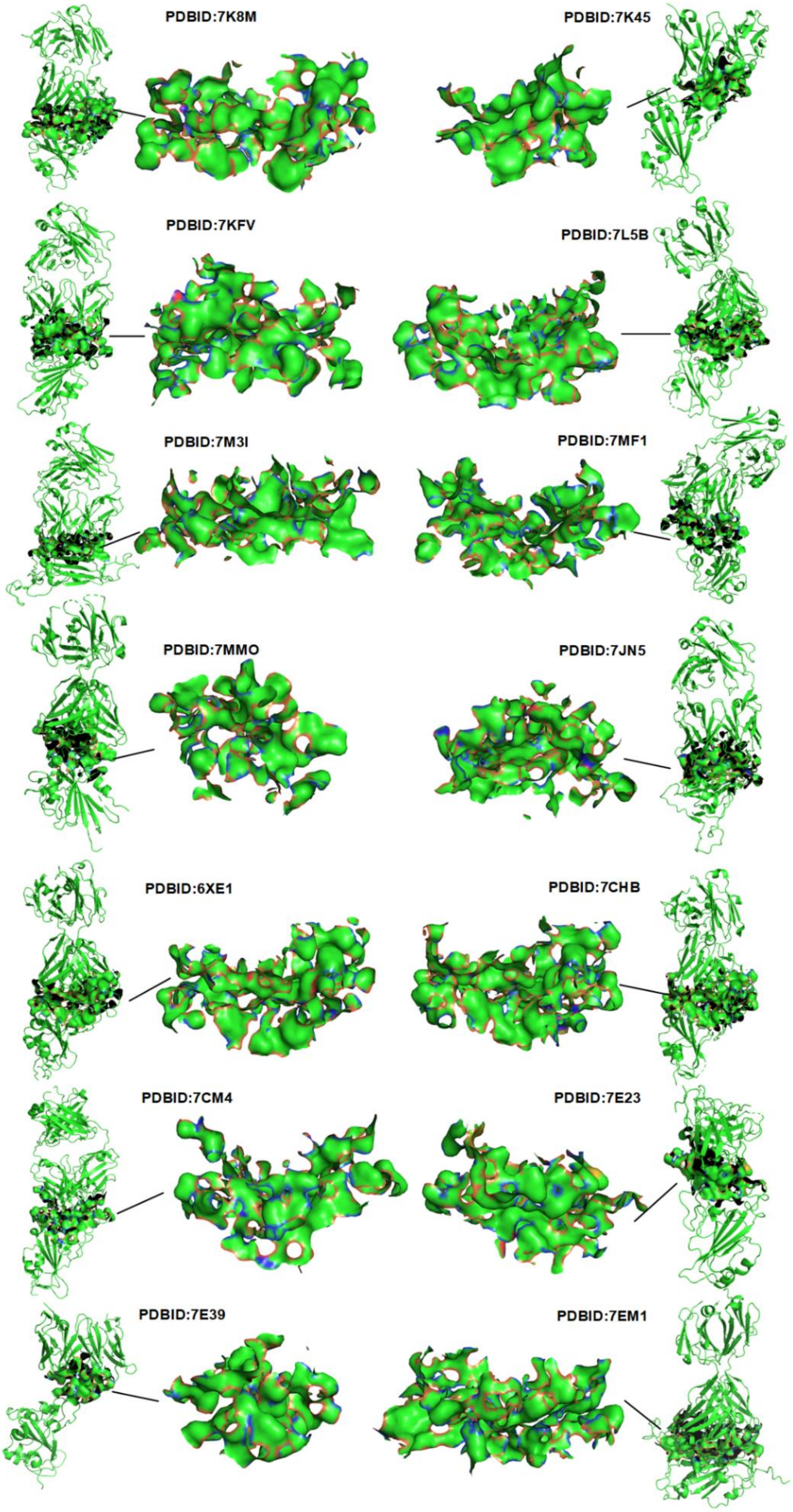

Figure S1 Strong hydrophobic-hydrophobic interactions at the interfaces of 14 RBD-antibody complexes.

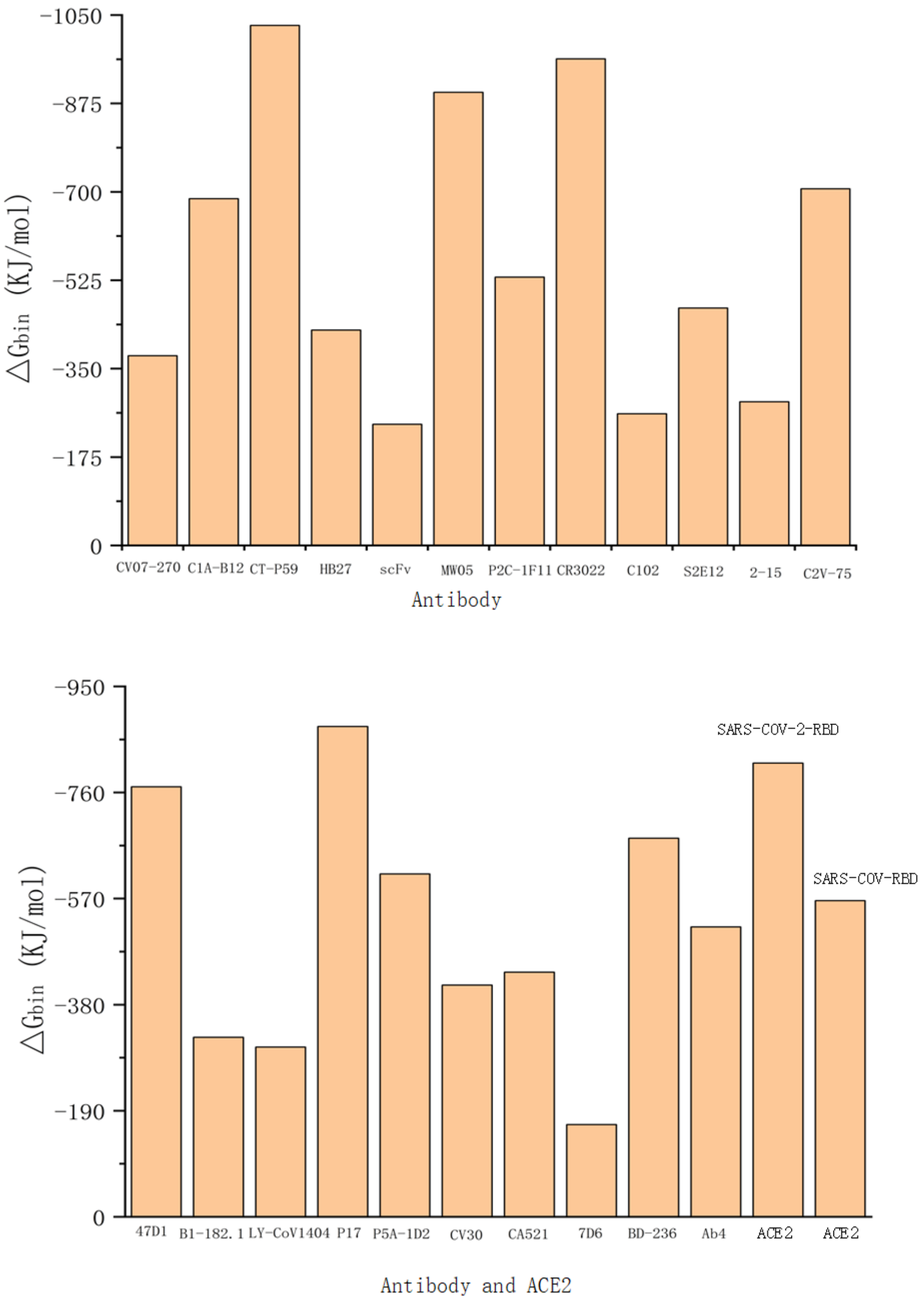

Figure S2 Binding energy (ΔG_{bin}) of the RBD-antibody complexes, the SARS-COV-S-RBD-ACE2 complex and the RBD-ACE2 complex.

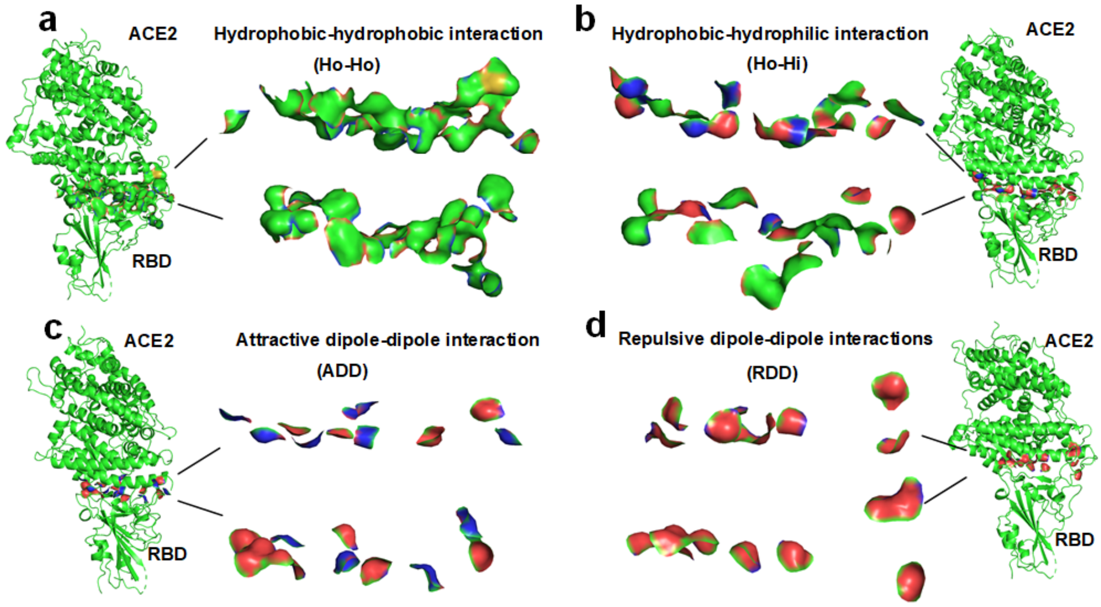

Figure S3 Four types of surface interactions in-between SARS-CoV-2 S RBD and ACE2 at the interface of the complex. The surface areas at the interface are spaced for demonstration.

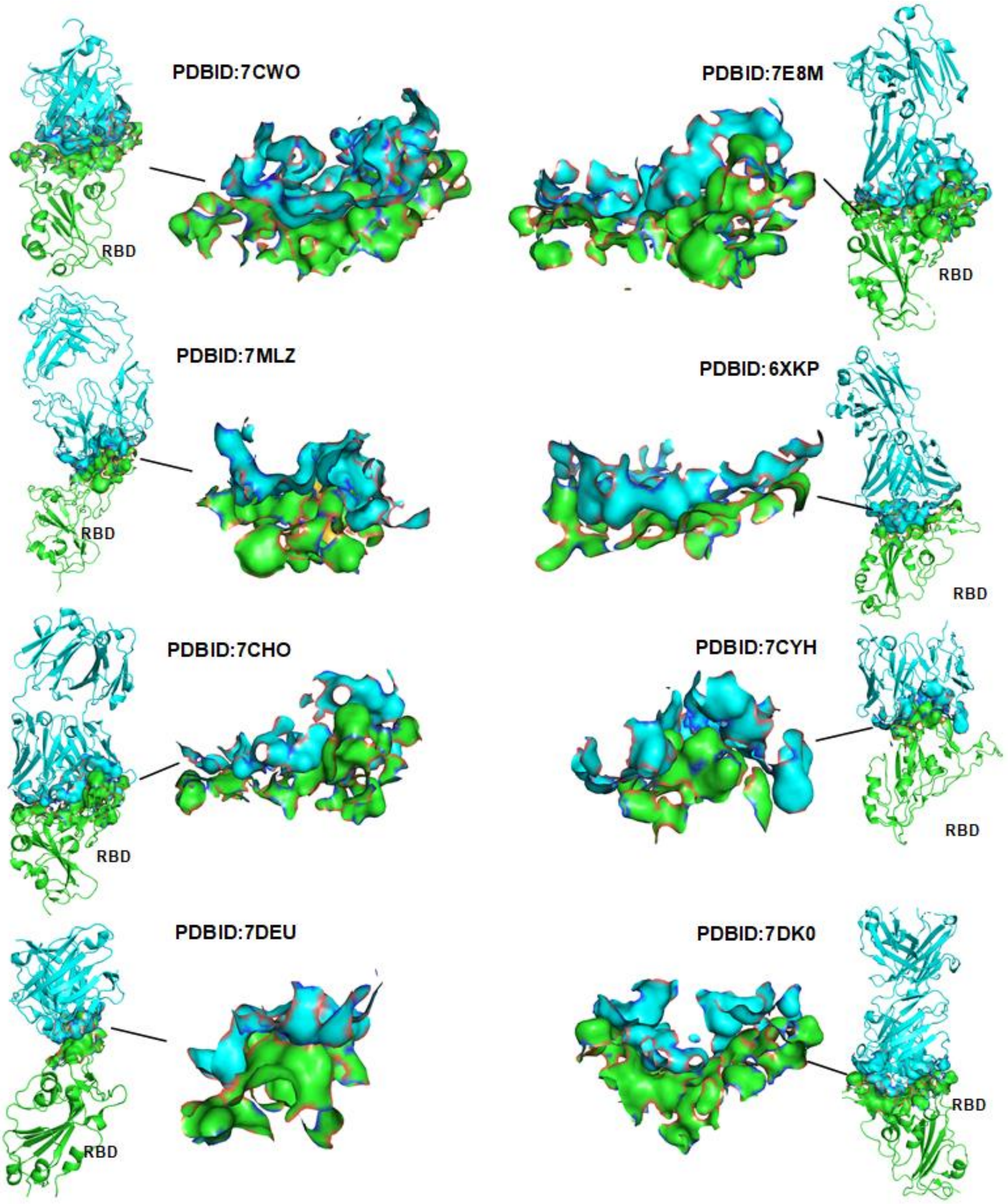

Figure S4 Strong hydrophobic-hydrophobic interactions at the interfaces of 8 RBD-antibody complexes. The structure of RBD and antibodies are colored in different colors.
